# Viability of African Swine Fever Virus with the Shallow Burial with Carbon Carcass Disposal Method

**DOI:** 10.3390/pathogens12040628

**Published:** 2023-04-21

**Authors:** Hoang Minh Duc, Mark Hutchinson, Gary A. Flory, Pham Hong Ngan, Hoang Minh Son, Le Van Hung, Tran Thi Khanh Hoa, Nguyen Thi Lan, Truong Quang Lam, Dale Rozeboom, Marta D. Remmenga, Matthew Vuolo, Robert Miknis, Amira Burns, Renée Flory

**Affiliations:** 1 Department of Veterinary Public Health, Faculty of Veterinary Medicine, Vietnam National University of Agriculture, Trau Quy, Gia Lam, Hanoi 12406, Vietnam; 2 Maine Food and Agriculture Center, University of Maine Cooperative Extension, Orono, ME 04473, USA; 3 G.A. Flory Consulting, Mt. Crawford, VA 22841, USA; 4 Department of Anatomy and Histology, Faculty of Veterinary Medicine, Vietnam National University of Agriculture, Trau Quy, Gia Lam, Hanoi 12406, Vietnam; 5 Faculty of Veterinary Medicine, Vietnam National Univeristy of Agriculture, Trau Quy, Gia Lam, Hanoi 12406, Vietnam; 6 Department of Pathoglogy, Faculty of Veterinary Medicine, Vietnam National University of Agriculture, Trau Quy, Gia Lam, Hanoi 12406, Vietnam; 7 Department of Animal Science, Michigan State University Cooperative Extension, Lansing, MI 48824, USA; 8 Center for Epidemiology and Animal Health, Veterinary Service, U.S. Department of Agriculture, Animal and Plant Health Inspection Services, Fort Collins, CO 80521, USA; 9 U.S. Department of Agriculture, Animal and Plant Health Inspection Services, Fort Collins, CO 80521, USA; 10 Department of Statistics, Colorado State University, Fort Collins, CO 80523, USA; 11 English Department, Johns Hopkins University, Baltimore, MD 21218, USA

**Keywords:** shallow burial with carbon, above ground burial, ASF virus, decomposition, inactivation

## Abstract

African swine fever (ASF) is a highly contagious swine disease with high mortality. In many countries, culling pigs infected and exposed to the ASF virus is mandatory to control the disease, which poses a real challenge in the disposal of large numbers of carcasses during ASF outbreaks. Shallow burial with carbon (SBC) Thanks ew mortality disposal method developed from deep burial and composting. The present study investigates the effectiveness of SBC in disposing of ASF virus-infected pigs. The real-time PCR results showed that DNA of the ASF virus was still detected in bone marrow samples on day 56, while the virus isolation test revealed that the infectious ASF virus was destroyed in both spleen and bone marrow samples on day 5. Interestingly, decomposition was found to occur rapidly in these shallow burial pits. On day 144, only large bones were found in the burial pit. In general, the results of this study indicated that SBC is a potential method for the disposal of ASF-infected carcasses; however, further studies are needed to provide more scientific evidence for the efficacy of SBC in different environment conditions.

## 1. Introduction

African swine fever (ASF) virus (ASFv) has been known as a highly contagious DNA virus of the *Asfarviridae* family, causing the most devastating swine disease, which results in socioeconomic losses for pork chain production [1]. Effective vaccines and treatments are not available yet for ASF, so the disposal of pigs infected or exposed to ASFv is one of the necessary measures to prevent the spread of the virus [2,3,4]. As a result, a large number of pig carcasses usually need to be disposed of safely during an ASF outbreak, which is a real challenge. In Europe, since 2017, ASF outbreaks led to the loss of 2.3 millions pigs [5]. In Russia and Eastern Europe, over 800,000 pigs were culled during 4 years (2014–2017) of an ASF outbreak [2]. In China, a total of approximately 1,193,000 pigs were destroyed during the 165 outbreaks that occurred from 2018 to March 2022. Approximately 6 million pigs were disposed of in Vietnam from February 2019 to May 2020 [6,7]. At present, deep burial, incineration, and composting are often used to dispose of animal carcasses in many countries around the world [8]. However, each of these methods has different advantages and disadvantages [8,9]. Deep burial is capable of disposing of a large number of carcasses, but the decomposition in the burial pit occurs slowly because oxygen is limited and the addition of lime inhibits the activity of microorganism community [10]. Likewise, the use of deep burial requires intensive labor and heavy equipment to dig deep pits and may also lead to the contamination of groundwater [11]. For incineration, the main advantage is its ability to rapidly inactivate pathogens by high temperatures, and the disadvantage is the need for fuel and incinerators [12,13]. Another disadvantage is the application of incineration may result in the production of dioxins, furan, odor, and aerosolize infective agents [12,14,15].

Composting is a natural process of decomposition based on the activity of aerobic microorganisms and has been recognized as a simple and economical method for disposal of animal mortality [10,16,17]. Therefore, this method has been effectively employed in many countries including Australia, New Zealand, the United States, and Canada for both the daily and emergency disposal of animal mortality [8,18]. Previous studies also demonstrated that composting could inactivate some common animal pathogens causing Foot-and-Mouth Disease virus (FMD) [10,19] and Porcine Epidemic Diarrhea (PED) virus [20]. However, there are still some concerns about composting related to the escape of pathogens and/or the release of odor from compost piles, as the carbon layer covering the compost piles can be easily disturbed by insects, scavengers, rats, and other animals. Heavy rain and flooding may also affect the stability of compost piles, thereby exposing animal carcasses to the environment [21]. In Vietnam, a number of infected pigs have been illegally thrown into inappropriate places such as rivers, lakes, and roadsides during the ASF outbreak in 2019, possibly due to the lack of convenient and economical disposal methods [22]. Therefore, a new disposal method is needed to manage animal carcasses during outbreaks, especially ASF outbreaks. Shallow burial with carbon material (SBC) may be an additional tool to assist responders in the disposal of ASF-infected animal carcasses. SBC is a hybrid between deep burial and composting. It involves digging a shallow trench, placing a carbon source such as rice hulls or saw dust in the bottom of the trench followed by a layer of carcasses, covering the carcasses with the excavated material, and finally seeding the mound. SBC has been implemented successfully at several sites within the United States and abroad [11,23]. This method has also been shown to be capable of inactivating several pathogens including the Seneca Valley virus and Swine Pox virus [21]. The aim of the present study was to investigate the viability of ASFv in swine carcasses disposed of with SBC.

## 2. Materials and Methods

### 2.1. Materials

**Figure 1:**
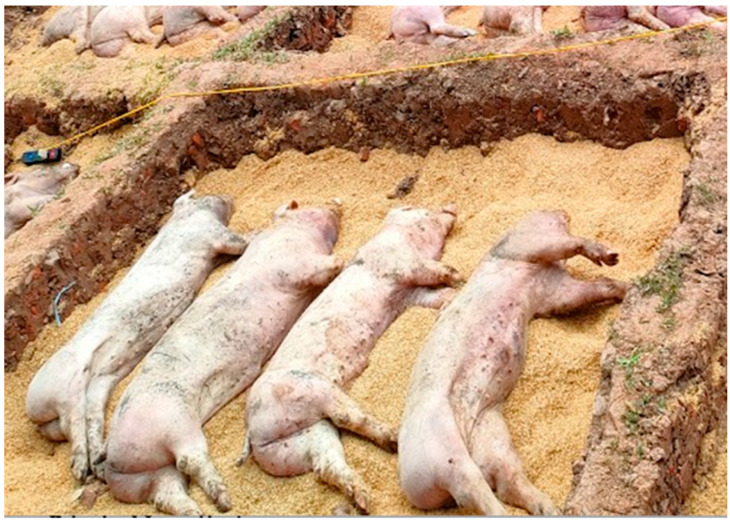
Four swine carcasses were placed in each SBC pit, facing the same direction.

Forty-eight pigs with typical symptoms of ASF were used for this study (Figure 1). Prior to burial, lymph nodes were also collected for the real-time PCR test to confirm the presence of ASFv. Rice hulls were collected from local farms near the experiment location.

### 2.2. Shallow Burial Pit Construction

In a biosecure area at the Veterinary Hospital, Vietnam National University of Agriculture (VNUA), 11 SBC pits, each containing 4 pigs infected with ASFv, were constructed. Briefly, SBC pits were dug with dimensions of 2 m wide × 2.5 m long × 55 cm deep (Figure 1). Approximately 30 cm of rice hulls was placed in the bottom of the pit (Figure 1). An analog thermometer lead was placed on top of the rice hulls in the center of the pit. Four market-size swine carcasses (60–80 kg) were then laid in the pit on their sides in a single layer as close together as possible, facing the same direction (Figure 1). The excavated soil was then returned to the pit and mounded in the center to shed water. Each SBC pit containing 4 pigs was designated for a specific day for excavation and data collection. Eleven pits corresponded to collection days 1, 3, 5, 7, 14, 21, 28, 35, 56, 144, and 288. The temperature of the burial pits was monitored daily using a handheld thermometer and data logger.

### 2.3. Sample Processing

**Figure 2:**
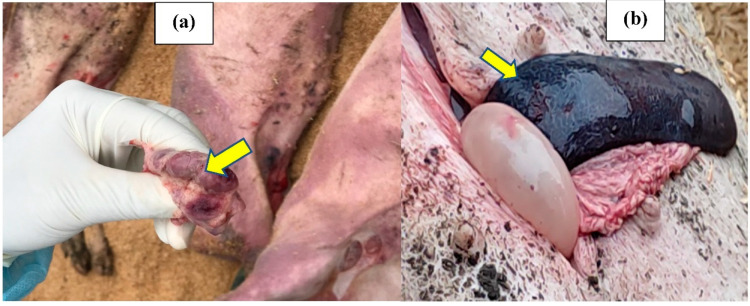
Pathology of ASF-infected swine carcasses used in this study. (**a**) Hemorrhagic lymph node. (**b**) Enlarged hemorrhagic spleen.

**Figure 3:**
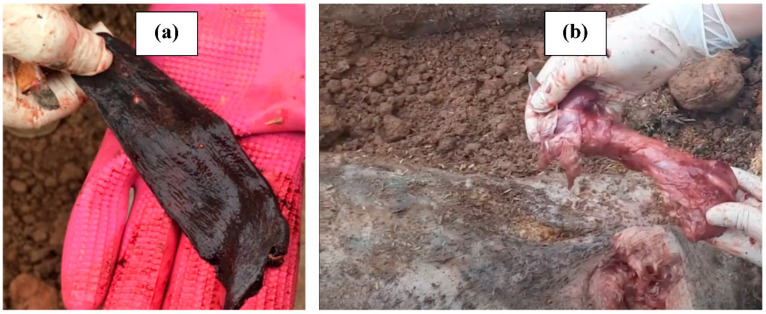
Spleen (**a**) and bone marrow (**b**) were extracted from buried swine carcasses.

On day 0, lymph node and spleen samples were collected from 4 pigs that were not buried for the detection and determination of ASFv concentration (Figure 2). Lymph node samples of 44 pigs buried in 11 pits were also collected before burying for the ASFv detection and isolation under the biosafety level 2 condition. After burial, the SBC pit was excavated on a specific day and samples (spleen and bone marrow) were extracted from carcasses and tested by PCR and cell culture to determine the survival of ASFv (Figure 3). Each sample was thoroughly homogenized in Phosphate Buffered Saline (PBS) using Retsch MM400 (Retsch, Germany) before it was centrifuged at 3000 rpm for 10 min. The supernatant was transferred to a new tube and stored at −80 °C for virus detection and isolation.

### 2.4. Detection of African Swine Fever Virus by Real-Time PCR

The DNA extraction of ASFv was performed using the MagMAX™ Viral/Pathogen Nucleic Acid Isolation Kit (Thermo Fisher, Waltham, MA, USA) following the manufacturer’s instructions. A real-time PCR was carried out according to the previously described method with TaqMan probe (5′-FAM-TTCCATCAAAGTTCTGCAGCTCTT-TAMRA-3′) and primer pair (forward, 5′-TGCTCATGGTATCAATCTTATCG-3′; Reverse, 5′-CCACTGGGTTGGTATTCCTC-3′) [24]. The PCR mixture (25 µL) contained 5 µL of nuclease-free water; 12.5 µL of PCR master mix 2X (Invitrogen superscript III qRT-PCR, Thermo Fisher, Waltham, MA, USA); 2.5 µL of primer—probe mix (forward primer (0.6 µM); reverse primer (0.6 µM); TaqMan probe (0.3 µM)); and 5 µL of DNA template. The mixture was then used for a real-time PCR using a CFX96-real-time PCR system under the following conditions: 1 cycle at 95 °C for 2 min, 45 cycles of 95 °C for 15 s, and 60 °C for 45 s.

### 2.5. Isolation of African Swine Fever Virus by Cell Culture

**Figure 4:**
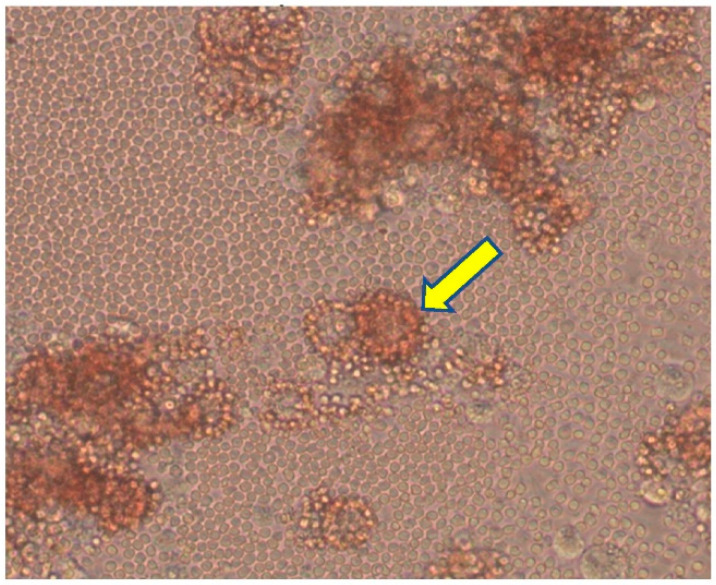
Hemadsorption in ASF virus-infected cells. Arrow indicates HAD rosettes.

For preparation, frozen Porcine Avelolar Macrophages (PAMs) were liquefied in a water bath at 37 °C. After that, the cell suspension was centrifuged at 2000 rpm for 10 min at room temperature. The supernatant was discarded, and the pellet was rinsed with 5 mL of PBS buffer. The washed pellet was resuspended in 10 mL of culture medium (RPMI 1640, 10% fetal bovine serum) to obtain the cell concentration of 5 × 10^6^ cells/mL. An aliquot of cell suspension was transferred into a flat bottom microplate (24 wells) and incubated for 16–24 h at 37 °C in 5% CO_2_. Subsequently, PAMs were infected with 100 µL of 10-fold serial dilutions of the prepared sample (4 wells for each dilution) and then incubated for 30 min. Hemadsorbing ASFv was used instead of the prepared sample in the positive control. For the negative control, wells were not inoculated with the sample. Following the incubation, suspension was discarded and 200 µL of fresh RPMI supplemented with 10% of fetal bovine serum, 1% antibiotic and antifungus, and 10% swine erythrocyte was transferred to each well and incubated at 37 °C in 5% CO_2_. The plate was monitored daily for 7 days under a microscope for the presence of the cytopathic effect (CPE) or hemadsorption (HAD) (Figure 4).

### 2.6. Bioassay

A bioassay was carried out to detect infectious ASFv in samples collected on days 28 and 56 that were PCR-positive but negative for virus isolation. A total of 10 healthy pigs of 20 kg (8 weeks old) purchased from an ASF-free farm were used for each experiment. The pigs were divided into a control and a treatment group. Pigs in each group (5 pigs) were housed in a pen of 9 m^2^ in a 16 m^2^ room of the Veterinary Hospital at VNUA. The rooms were environmentally controlled with an air conditioner and ventilator. Femur bone marrow from 4 carcasses from each sample time (day 28 and day 56) was pooled to make the supernatant. After 3 days of adaptation after arriving to the pen, each pig in the treatment group received 1 mL of intramuscular injection of the supernatant. Pigs in the control group were injected with 1 mL of Ringer’s lactate. The pigs were then monitored daily for clinical signs of ASF. On day 0, 3, 4, 5, 6, 10, and 25 after injection, blood samples were collected for the detection of ASFv using a real-time PCR as described above.

### 2.7. Statistical Methodology

There are some important assumptions associated with statistical methodology. Virus concentration was assumed to decay in a straight line on the log10-scale over time. While the observed data above the cell culture detection limit appear to support this assumption, the true shape of the regression line was difficult to assess with only 3 sampling days featuring concentrations above the detection limit. Virus concentration below the cell culture detection limit was also assumed to decay in a straight line on a log10-scale as a continuation of the model calculated for the data observed above the detection limit. Violation of these assumptions could lead to a bias in the results presented in this paper, particularly if there was a curve in the true virus concentration decay as it approached zero. The 2 and 3 data points below the cell culture detection limit on day 3 for the spleen and femur samples, respectively, were set equal to 10^2^ HAD50, the cell culture detection limit. This was a conservative estimate because 10^2^ HAD50 was the maximum concentration possible without detection by the cell culture test. Setting these data points to a fixed value artificially reduced the variance of virus concentrations for that sampling day and for the models. Thus, the average time to reach 1 HAD50 may be artificially increased, while the prediction interval widths may be artificially decreased. An additional assumption made in the sampling design was that the variance of virus decay between different pits was approximately 0, which justified the use of only 1 pit for each sampling day. Factors held constant in the study design that could affect the virus decay in real-world scenarios included weight and age of the pig carcasses, soil type, starting ambient soil temperature, weather conditions, and the quality of the SBC pit construction.

## 3. Results

### 3.1. Temperature Profile

**Figure 5:**
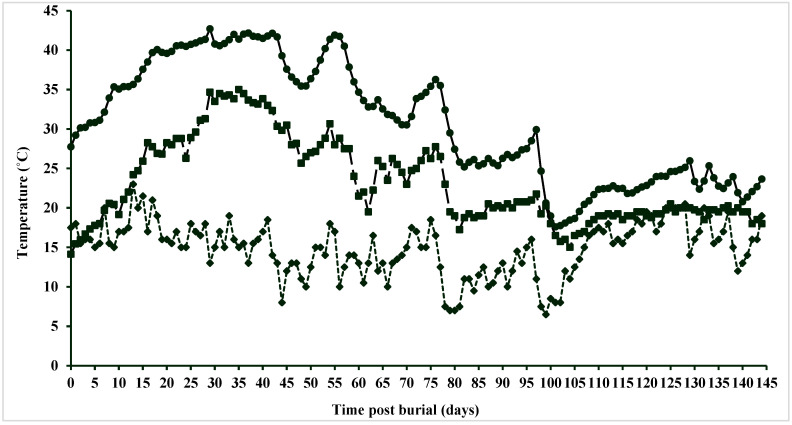
Temperature profile of SBC pits. Square dot line (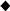), long dash line (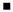), solid line (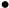) is air tempearture, soil temperature, and core temperature, respectively.

Figure 5 reveals the temperature profile of the SBC pits. The soil and core temperature increased gradually, reaching 34.67 °C and 42.70 °C after 29 days of SBC pit establishment, respectively. However, the air temperature recorded on day 29 was 13 °C. Overall, the soil temperature of the SBC pit showed the same trend as that of the core temperature but was approximately 10 °C lower. The core temperature was maintained above 40 °C from day 22 to day 43 before gradually going down but was still higher than the soil and air temperature throughout the experiment. The soil temperature remained higher than the air temperature until day 108. From day 109 to day 144, the soil temperature and air temperature were quite similar and ranged from 12 °C to 20.5 °C.

### 3.2. Detection and Viability of African Swine Fever Virus

**Figure 6:**
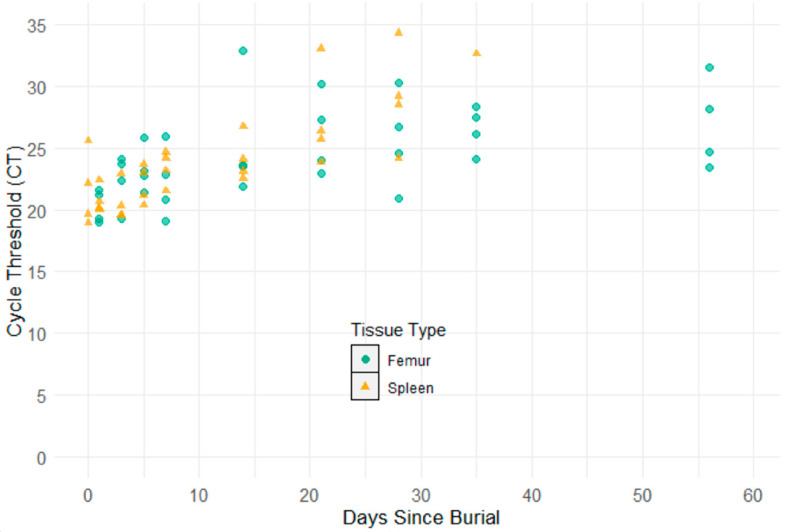
Scatter plot of Ct of post-burial spleen and femur bone marrow samples. Green dots are the Ct values of femur bone marrow samples. Orange triangles are the Ct values of spleen samples.

The DNA of ASFv was detected in all 48 pigs in this study on day 0. Pre-burial, the Ct value of the lymph node samples of 48 pigs ranged from 18.02 to 26.28. The DNA of ASFv was detected in the spleen and bone marrow samples of all carcasses after burial for days 1 through 21, and on day 28, DNA of the virus was still detected in all spleen (Ct from 24.15 to 34.31) and bone marrow samples (Ct from 20.92 to 30.29) (Figure 6). However, on day 35, only one spleen sample had detectable DNA of ASFv (Ct = 32.63), while all bone marrow samples tested positive for ASFv DNA on day 35 (Ct from 24.12 to 28.34). On day 56, the DNA of ASFv was not detected in any spleen samples. On the contrary, the results of the real-time PCR test revealed that the DNA of the virus was found in all bone marrow samples on day 56 with Ct values ranging from 23.46 to 31.52 (Figure 6).

**Figure 7:**
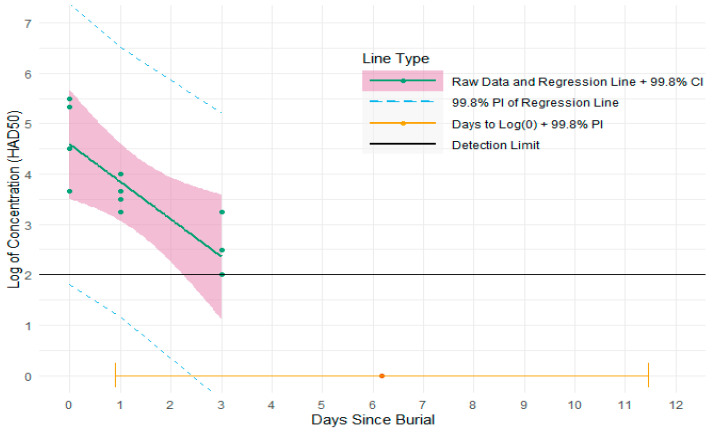
Plot of log_10_ of the ASFv concentration for spleen samples tested with 99.8% confidence and prediction intervals. Green dots are the raw cell culture data. The green line is the best fit simple linear regression line for log_10_ of the ASFv concentration regressed on days since burial. The pink shaded area is the 99.8% confidence interval for the best fit regression line. The dashed blue lines represent the lower and upper bounds of the 99.8% prediction interval for the plotted regression line. The orange dot is the estimated mean number of days to log_10_(0) ASFv concentration, equivalent to 1 ASFv particle. The orange line or interval around the orange dot is the 99.8% prediction interval for the estimated number of days to log_10_(0) ASFv concentration.

**Figure 8:**
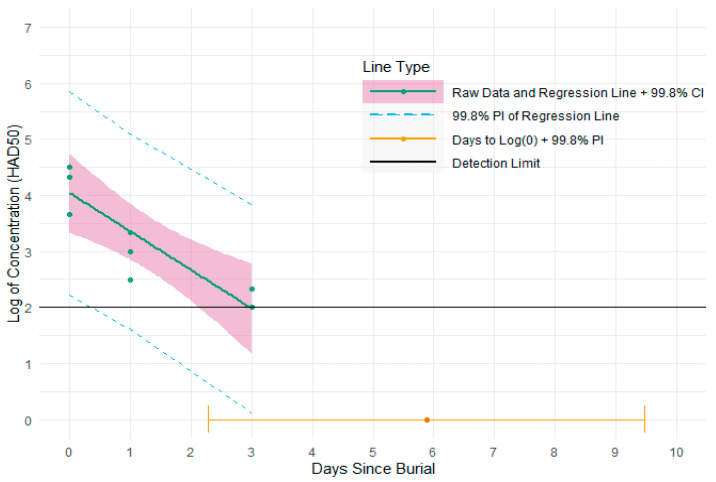
Plot of days since burial vs. log_10_ of the ASFv concentration for femur bone marrow samples tested with 99.8% confidence and prediction intervals. Green dots are the raw cell culture data. The green line is the best fit simple linear regression line for log_10_ of the ASFv concentration regressed on days since burial. The pink shaded area is the 99.8% confidence interval for the best fit regression line. The dashed blue lines represent the lower and upper bounds of the 99.8% prediction interval for the plotted regression line. The orange dot is the estimated mean number of days to log_10_(0) ASFv concentration, equivalent to 1 ASFv particle. The orange line or interval around the orange dot is the 99.8% prediction interval for the estimated number of days to log_10_(0) ASFv concentration.

The virus isolation test showed that the titer of ASFv recovered from the lymph node samples of 48 pigs prior to burial varied from 10^3.3^ to 10^5.7^ HAD50, with the majority ranging from 10^4.3^ to 10^4.7^ HAD50. The virus concentration from the spleen samples of four pigs before burial (day 0) varied from 10^3.7^ to 10^5.5^ HAD50. On day 1, the infectious virus was detected in all four spleen and bone marrow samples (from exhumed pigs) with the concentration varying from 10^3.2^ to 10^4^ HAD50 for spleen samples and from 10^2.5^ to 10^3.3^ HAD50 for bone marrow samples. By day 3, the infectious ASFv particles were only detected in two of four spleen samples with titer from 10^2.5^ to 10^3.3^ HAD50 and one of four bone marrow samples (10^2.3^ HAD50) (Figure 7 and Figure 8). Five days post-burial, ASFv was found to be completely inactivated in all spleen and bone marrow samples.

For the spleen tissues, the mean of virus concentration in the population was estimated to decay to 1 HAD50 in 6.2 days with 999 out of 1000 pigs reaching 1 HAD50 by 11.5 days (Figure 7; Table 1). For the femur bone marrow, the mean of virus concentration in the population was predicted to degrade to 1 HAD50 in 5.9 days with 999 out of 1000 pigs reaching 1 HAD50 by 9.5 days (Figure 8; Table 1).

For the bioassay tests, negative virus isolation for samples collected on day 28 and samples collected on day 56 were each confirmed in 10 healthy pigs. Twenty-five days after inoculation, typical clinical signs of ASF were not observed in any tested pigs. The result of the PCR also showed that the ASF virus was not detected in blood samples collected on day 0, 3, 4, 5, 6, 10, and 25 post-injection.

### 3.3. Carcass Decomposition

**Figure 9:**
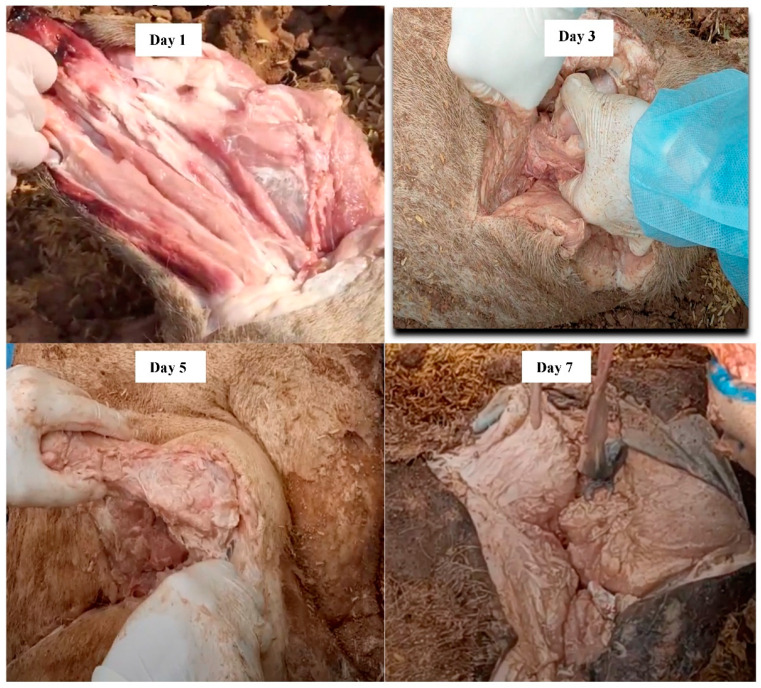

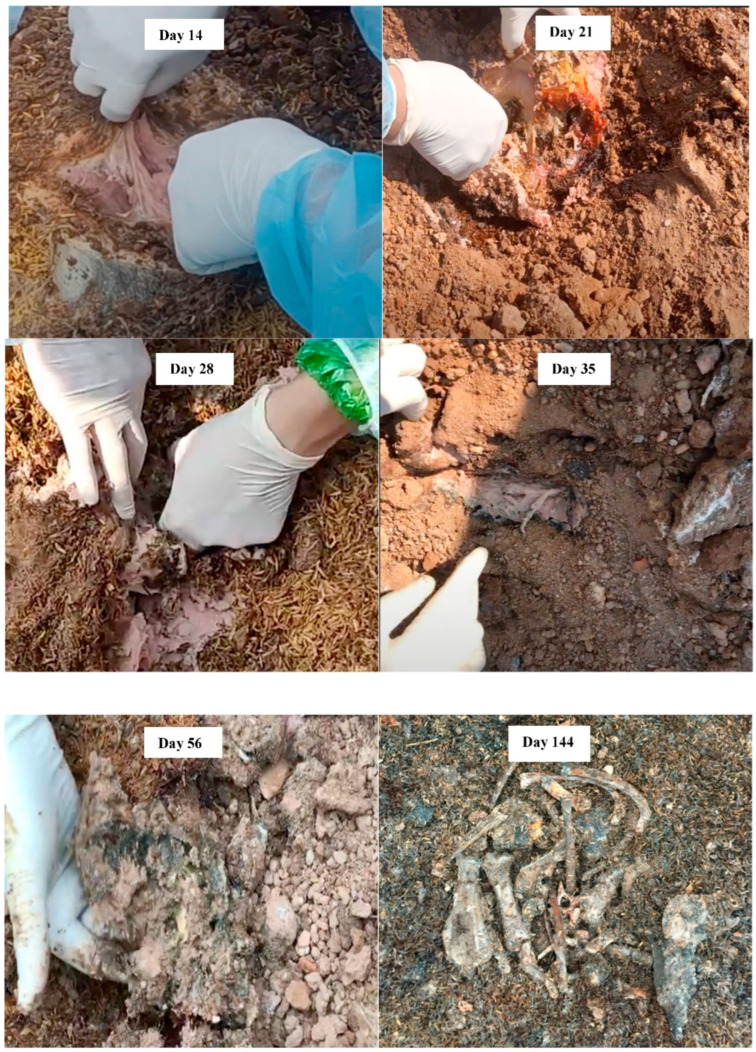
The decomposition of carcasses in SBC pits at day 1, 3, 5, 7, 14, 21, 28, 35, 56, and 144.

On day 1, the muscle tissue still looked fresh with color similar to that of day 0 (Figure 9). From day 3 to 7, the color of the muscle tissue started changing to a gray color and there was a strong smell during the carcass exhumation. On day 14, whole carcasses were partly degraded, and muscle tissue was easily detached from bones and had turned white. Organs had turned black by day 14. From day 21 to 56, the carcasses continued to decompose rapidly, thereby the abdomen and shape of the swine body became difficult to recognize. In addition, the smell reduced gradually during exhumation between day 21 and 56. On day 144, whole carcasses were almost completely decomposed, and the smell was not recognized during exhumation. Although large bones still remained, they had become spongy, especially the rib bones (Figure 9).

## 4. Discussion

Despite the advances in vaccine and biosecurity measures, animal diseases still break out all over the world. In the event of an outbreak caused by contagious pathogens, the depopulation of infected/exposed animals and management of carcasses play a very important role in preventing the spread of infectious diseases [8]. The disposal of animal mortality is a challenge for livestock farming systems in outbreaks but has become routine as a large number of animal carcasses are generated daily from a global livestock population of approximately 1.9 × 10^10^ birds and 2.31 × 10^8^ mammals [13].

Deep burial and composting are the most-used methods for the disposal of animal carcasses [8]. The main advantage of deep burial is its capacity to dispose of a vast quantity of carcasses. However, it requires heavy equipment or excessive labor to dig deep burial pits [11]. Deep burial is not suitable for the areas that have high groundwater and/or are close to the water sources as it can cause water contamination and difficulties in digging burial pits [25]. The pathogens present in carcasses are assumed to be able to survive for a long time in a deep burial pit due to the suitable environment such as a low temperature and anaerobic condition. To eliminate the pathogens in carcasses, lime is usually added to deep burial pits. However, this inhibits microorganism activities, consequently slowing down the decomposition process of carcasses and limiting future land use [11]. A previous study showed that the carcass decomposition was not complete even after 3 years [10]. Composting has proved to be effective in destroying some common animal pathogens, such as Foot-and-Mouth Disease virus (FMD) [10,19], Porcine Epidemic Diarrhea (PED) virus [20], and ASF virus [26]. However, the need for a large amount of carbon material hinders the use of this method in some areas during an outbreak. In addition, pathogens present in carcasses may escape from compost piles via insects, scavengers, rats, and other animals as well as in some special weather conditions such as heavy rain and/or flooding [8,13].

SBC is a newly developed carcass disposal method based on the advantages and disadvantages of deep burial and composting [11]. When compared to deep burial, SBC facilitates the activities of aerobic microorganisms, thereby accelerating the carcass decomposition process [23]. The results of our study also support the idea that carcasses rapidly decompose in an SBC pit. By day 144, most of the tissues were completely decomposed (Figure 9). Although large bones still remained, they had become spongy, especially the rib bones. Furthermore, it is much more economical and easier to establish SBC pits in comparison with deep burial pits [11]. The major advantage of SBC over composting is the requirement of a smaller volume of carbon material, indicating the cost-effectiveness of SBC. For SBC, carcasses are placed in the burial pit and covered with soil, while they are placed on the ground and covered with carbon material for composting. Thus, it is more difficult for insects, scavengers, rats, and other animals to gain access to carcasses in SBC pits than in compost piles. SBC has also been considered to achieve a higher biosecurity level compared to composting during heavy rain and flooding. In general, each disposal method has different strong points. As a result, selection of the disposal method should be based on the specific conditions of farms, areas, and country regulation even in specific cases. With several advantages as mentioned above, SBC is expected to provide a potential tool for carcass management.

A previous study has demonstrated the effectiveness of the SBC method in managing sheep carcasses infected with Peste des Petits Ruminants virus (PPRv). However, the survival of the PPRv in sheep carcasses was not investigated [23]. Ebling et al. [21] determined the viability of Swinepox virus (SPv) in bone marrow during SBC on day 7, 14, 21, 28, and around months 2, 3, 6, and 12 post-burial. Although the SPv DNA was detected in 93% of 160 bone marrow samples, the viable virus was only recovered from 11 (55%) bone marrow samples collected on day 7. A statistical analysis estimated that the virus was completely inactivated around day 11 post-burial. Another shallow burial trial was conducted by Zani et al. [27]. In that study, bone marrow samples were extracted from wild boar carcasses naturally infected with ASFv in various locations and time points from 18 to 440 days for virus detection and isolation. The results showed that virus DNA was found in 17 out of 20 burial sites. However, the virus was not isolated in all samples tested. Fischer et al. [28] determined the stability of ASFv in carcasses of domestic pigs and wild boar experimentally infected with ASFv. The findings of that study showed that ASFv DNA was still detected in bones stored for 3 months at room temperature, while the infectious virus was not recovered after a week of storage. The results of our study are consistent with the previous studies mentioned above. It was found that all spleen and bone marrow samples tested positive for ASFv DNA on day 28. However, none of the samples was positive with virus isolation on day 5. The statistical analysis also suggests that ASFv in the spleen and femur bone marrow was completely destroyed in 6.2 days and 5.9 days, respectively. It is worth noting that the initial virus titer in the samples may affect the longevity of the virus. In the present study, relatively high initial virus titers (10^3.3^ to 10^5.5^ HAD50) were determined in the lymph node and spleen of pigs used for the experiment. Bioassays were used to examine the infectivity of the virus by administration of the suspicious samples to healthy pigs. This method is known to be more sensitive than virus isolation using the cell culture technique [29]. Previous studies demonstrated that pigs were infected with ASFv after feeding with samples that previously tested negative in virus isolation [29,30]. In this study, the result of the bioassay was in agreement with that of virus isolation, confirming that ASFv was inactivated in samples collected on day 28 and 56.

Temperature has been suggested as the main contributor for virus inactivation in composting. Guan et al. [17] reported that a compost temperature of 50–55 °C was enough to kill both Avian Influenza (AI) and Newcastle Disease (ND) viruses. Elving et al. [16] obtained similar results, showing that the AI virus was inactivated during composting at both mesophilic (35 °C) and thermophilic levels (45 and 55 °C). The study by Guan et al. [31] revealed that Bovine Viral Diarrhea (BVD) virus was destroyed at a compost temperature of 41 °C. Vitosh-Sillman et al. [20] investigated the survival of the PED virus in swine carcasses during composting; the findings showed that the PED virus was inactivated at 37 °C within 24 h in a compost windrow. As expected, the SBC temperature observed in this study was not as high as the compost temperature [26]. However, the temperatures obtained in the study were still higher than those achieved in a previous study conducted by Ebling et al. [21] on SBC. In our study, temperatures above 40 °C were maintained for more than 3 weeks in a burial pit, while temperatures obtained in the study of Ebling et al. [21] were less than 37 °C throughout the experiment. On the contrary, air temperatures recorded in this study were lower than those observed in the study of Ebling et al. [21] (Figure 5). It is important to note that ASFv was considered as a heat-sensitive virus that was destroyed at 37 °C after around 20 days [32]. Therefore, the temperature (>40 °C) obtained in our study could be an important contributor for ASFv inactivation.

Although temperature is often considered a key factor in pathogen inactivation, it is just one of many factors leading to successful inactivation. Within an SBC system, pathogen inactivation is likely a result of a combination of factors, including predation by microbial populations, shifts in pH, or the absence of a living host [28,33,34]. The specific environmental conditions in each burial site, including air temperature, rainfall, soil biochemical characteristics, and local worm activity, can be other factors affecting the length of virus viability [27,28,35]. Additionally, different carcass tissues may influence virus tenacity [28,34]. Likewise, the inactivation speed may partly depend on the characteristics of each virus [36]. Among the factors mentioned above, microbial activity seems to be the most important factor for virus inactivation, and it can be optimized by adjusting the depth of the SBC pit and carbon layer. As in composting, elevated temperatures have been recognized as an indicator of the microbial activity which likely plays an important role in pathogen inactivation [20,37,38]. As seen in Figure 5, the temperatures in our study increased gradually and reached a peak of 42.7 °C on day 29. The temperature then still maintained above 40 °C until day 43 before gradually reducing afterwards. Furthermore, the temperature in the core of the SBC system in the present study remained 5 to 10 °C higher throughout the experiment compared to the ambient soil temperature. The elevation of temperature and the difference in temperature of the core and soil in this study can be attributed to the metabolic activity of the microbial population within the system. This indicates that the SBC pits were well-established in the present study. Furthermore, the elevation of temperature was not notable in the study of Ebling et al. [21], suggesting that microbial activities in our study may be stronger than those in the study of Ebling et al. [21]. In the present study, the lymph node, spleen, and femur bone marrow tissues were selected to determine the tenancy of ASFv since it has been previous reported that these tissues contained high titers of ASFv as well as cells for the replication of ASFv [21,28]. Moreover, the femur bone was assumed to be able to protect ASFv from heat and other adverse conditions.

In summary, the findings of our study suggest that SBC is a potential method for the disposal of ASFv-infected swine carcasses. Interestingly, the ASFv in spleen and bone marrow samples was rapidly inactivated after 5 days of burial in the specific conditions of this study. In addition, the whole carcasses were found to be almost decomposed after 144 days. However, further studies are needed to validate the effectiveness of SBC in managing ASFv-infected carcasses in different environment conditions.

## Figures and Tables

**Table 1:** Prediction of ASFv inactivation.

Tissue	Mean Number of Days to 1 HAD50	Prediction Interval Confidence (%)	Number of Days to 1 HAD50
Spleen	6.17	95	3.33–9.01
		98	2.65–9.69
		99.8	0.89–11.45
Bone Marrow	5.88	95	3.94–7.81
		98	3.48–8.28
		99.8	2.28–9.48

## Data Availability

The data that support the findings of this study are available from the corresponding author upon reasonable request.
